# Laparoscopic treatment of triple ureteral with ureteral calculus: A case report

**DOI:** 10.1097/MD.0000000000043031

**Published:** 2025-06-27

**Authors:** Longyuhe Yang, Xuanfan Hu, Yueqiang Wang, Zhen Ma, Yu Tian, Yunliang Zhao, Jianbing Yang, Zhigang Zhang, Qiuyi Lu

**Affiliations:** a Urology Department, Southern Central Hospital of Yunnan Province (First People’s Hospital of Honghe State), Mengzi, China; b Ultrasonography Department, Southern Central Hospital of Yunnan Province (First People’s Hospital of Honghe State), Mengzi, China; c Anesthesiology Department, Southern Central Hospital of Yunnan Province (First People’s Hospital of Honghe State), Mengzi, China.

**Keywords:** laparoscopic, ureteral tailoring, ureteral triplication

## Abstract

**Rationale:**

Ureteral triplication (UT) is a rare congenital urinary tract anomaly, with approximately 100 cases reported globally, arising from abnormal branching of the ureteric bud during embryogenesis.

**Patient concerns:**

A 48-year-old male presented with recurrent left flank and abdominal pain for 3 years, worsening over the past year, accompanied by gross hematuria and purulent urine, suggesting urinary tract infection and obstruction.

**Diagnoses:**

Computed tomography urography and cystoscopy confirmed left-sided ureteral triplication, with 1 malformed ureter containing a calculus and proximally occluded, and another merging with the normal ureter.

**Interventions:**

Laparoscopic surgery was performed to excise 2 malformed ureters (1 opening into the prostate and the other merging with the normal ureter), followed by reconstruction of the normal ureter and intraoperative placement of a double-J stent.

**Outcomes:**

Postoperatively, no urinary leakage or infection occurred. Three-month follow-up demonstrated patent ureters, resolution of symptoms, and computed tomography urography-confirmed anatomical restoration.

**Lessons:**

Laparoscopic management is effective for complex ureteral anomalies, offering minimal invasiveness and rapid recovery. Individualized surgical strategies are critical for optimizing patient outcomes.

## 1. Introduction

Ureteral triplication (UT) is a rare upper urinary tract anomaly. Triple renal duplication was first described in 1870,^[1]^ and fewer than 100 cases of UT have been reported.^[2]^ Surgical intervention typically involves the removal of the poorly functioning duplicated renal segment and the ureter. In some cases, when the duplicated renal segment is sufficiently functional, bladder-ureter reimplantation or ureteral anastomosis may also be performed. Unlike previously reported cases, the 2 abnormal ureters in this patient were not connected to the renal pelvis, forming blind ends proximally; 1 opened into the normal ureter, while the other opened into the prostate, with the presence of calculi. We present a case of successful laparoscopic treatment of this patient with left-sided ureteral triplication.

## 2. Case presentation

**Figure 1.**
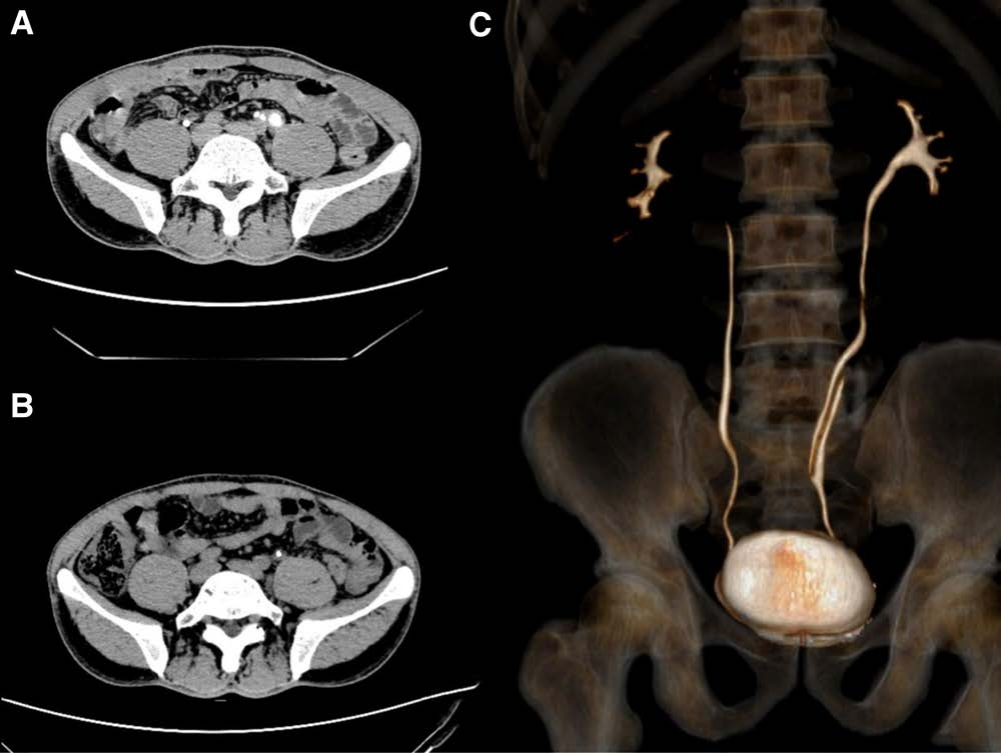
(A) Preoperative CTU indicating a left-sided triple ureter. (B) Three months postoperatively, a follow-up CTU shows patency of the ureters. (C) Left-sided ureteral malformation.

In July 2024, a 48-year-old male patient presented to our hospital with complaints of left flank and abdominal pain without any identifiable cause for the past 3 years. One year prior, the symptoms worsened, accompanied by gross hematuria and purulent urine. The patient underwent computed tomography urography and other examinations at a local hospital, which suggested a left ureteral cyst and referral to a higher-level hospital was recommended. Subsequent examinations and treatment were performed at our facility. Preoperative CT indicated a duplicated left ureter (Fig. 1). Urinalysis revealed signs of infection, whereas blood tests and biochemical examinations revealed no abnormalities. We scheduled the patient for surgery after 2 consecutive negative urine cultures were obtained.

**Figure 2.**
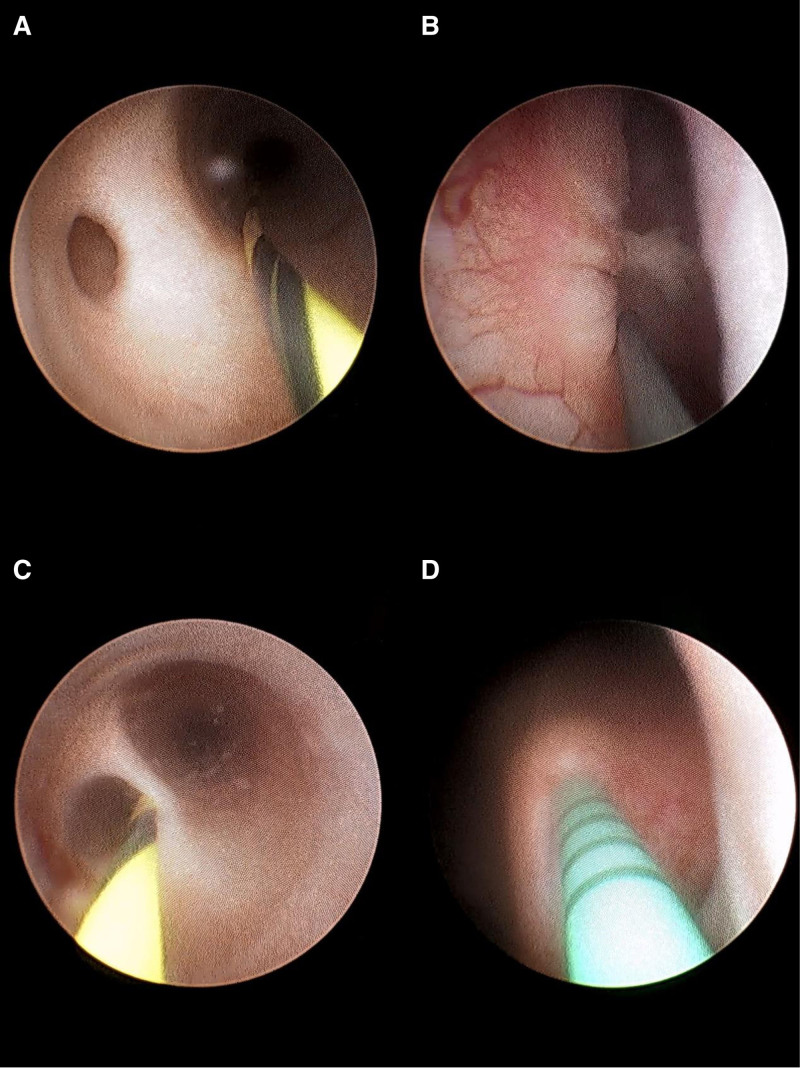
(A and B) During ureteroscopy, dual ureteral orifices were found, with significant obstruction in the upper segment of the larger ureter. (C and D) The other orifice is a normal ureter, and an F5 double-J stent was placed over a guidewire for intraoperative identification.

After anesthesia was administered, the patient was placed in the lithotomy position. A ureteroscope was used to examine the urethral, prostate, and bladder cavities, revealing no significant abnormalities. Under guidewire assistance, we accessed the left ureter and advanced approximately 10 cm to find 2 ureters, one of which was dilated. Continuing with the scope through the dilated ureter for an additional 6 cm, an occluded lumen with white floating debris was observed. The scope was then withdrawn and reintroduced through the other ureter into the renal pelvis, where an F6 double-J stent was placed (Fig. 2).

**Figure 3.**
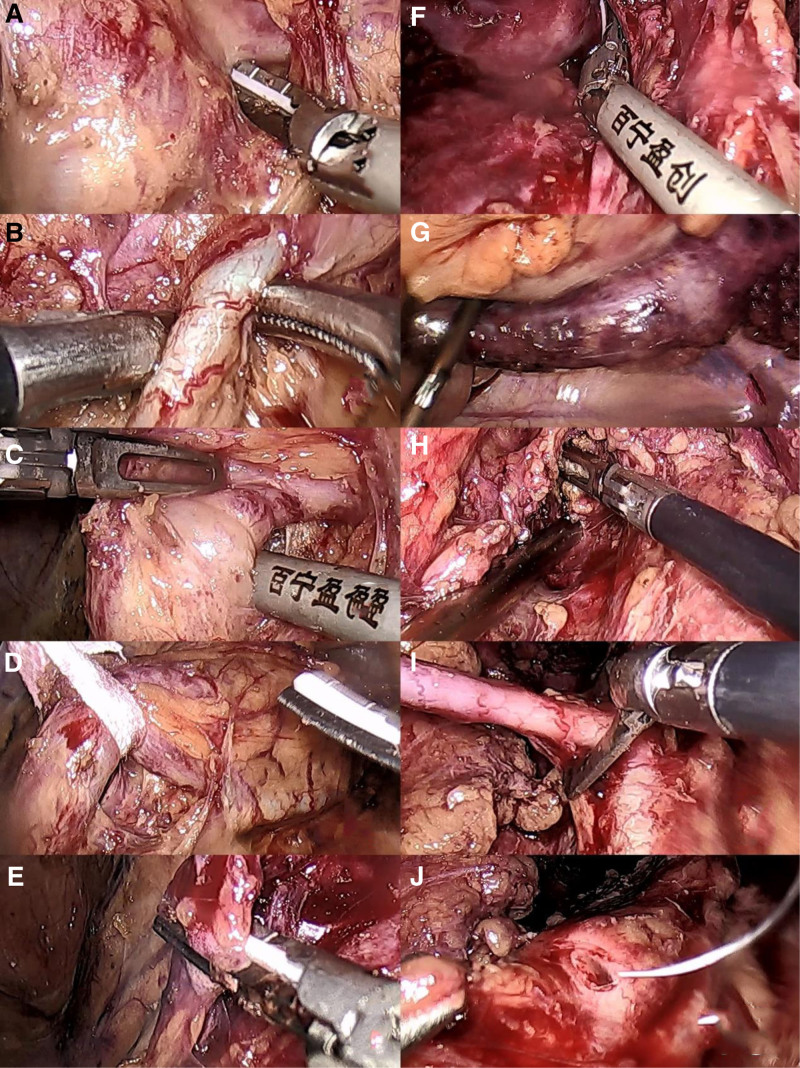
(A) Incision of the iliac vessels and separation of the peritoneum above. (B) Dissection of the normal ureter, revealing the previously placed double-J stent. (C) Dissection of the abnormal ureter. (D) Using gauze to separate the normal and abnormal ureters. (E) Cutting the proximal end of the abnormal ureter. (F) The first orifice of the abnormal ureter is located at the left prostate region. (G) The excised ureter contains calculi. (H) Clamping of the spermatic cord to control bleeding. (I) Free excision of the second duplicated ureter, trimming the junction with scissors. (J) Intermittent suturing of the ureteral lateral wall with 4-0 absorbable suture.

The patient was then placed in the right lateral decubitus position, with the head lower than the feet. After establishing pneumoperitoneum, an incision was made at the upper margin of the umbilicus, and the abdomen was elevated. A 10 mm trocar was inserted into the abdominal cavity, and the position was confirmed using a laparoscope. About 5 and 12 mm trocar channels were established lateral to the rectus abdominis muscles at the level of the umbilicus. An ultrasonic knife was used to release adhesions of the sigmoid colon, and the peritoneum was incised in the left iliac vascular region. Above the iliac vessels, the duplicated malformed ureter was dissected, revealing 3 ureters: 1 dilated malformed ureter with the upper end occluded at the ureteropelvic junction and the lower segment opening into the left prostate, measuring approximately 16 cm and containing calculi; and another malformed ureter slightly larger than a normal ureter, measuring approximately 6 cm, with the upper end occluded at the normal ureteropelvic junction and the lower end merging with the normal ureter into a single ureter. The 2 duplicated malformed ureters were completely excised, and the junctions were trimmed with scissors. The sidewall of the ureter was sutured intermittently using 4-0 absorbable sutures. Hemostasis was achieved in the vas deferens area using a home-lock clamp (Fig. 3). The surgical field was inspected for active bleeding, and a pelvic drain was placed. The trocars were removed, the drain was secured using sutures, and the incisions were closed. The surgery was successful, and the patient returned to the ward.

No antibiotics were administered postoperatively. The abdominal drain was removed on the second day after surgery, and the ureteral catheter was removed on the third day with no urinary leakage or urinary tract infection. Follow-up examinations 1 month later showed good results, with normal urinalysis and no bacterial growth in the cultures. The patient experienced smooth urination, and the left ureteral stent was removed during outpatient cystoscopy. Follow-up computed tomography urography 3 months after stent removal confirmed the patency of the ureter, with no abnormalities noted (Fig. 1).

## 3. Discussion and conclusion

In the fourth week of pregnancy, the ureteric bud arises from the mesonephric duct and comes into contact with the metanephric mesenchyme, inducing branching and division of the ureteric bud. Variations or defects in this branching process can lead to abnormalities. Theoretically, the formation of multiple ureteric buds may result in duplicated ureters. Ureteral quadruple malformations are extremely rare, with only approximately 10 cases reported in the literature.^[3]^ Additionally, there is 1 case report of a quintuple ureter involving a 3-year-old child who was born 60 kilometers from Chernobyl 24 years after the nuclear disaster.^[4]^ Other anomalies commonly associated with ureteral triplication included contralateral ureteral duplication (37%), ectopic ureters (28%), and renal dysplasia (8%). Occasionally, ureteral cysts, reflux urinary tract infections, vascular malformations, and genital malformations may also be found. Literature searches indicate that approximately 100 cases of UT have been reported globally, with Campbell identifying only three cases among over 47,000 autopsies.^[5]^

In the Smith classification,^[6]^ ureteral triplication is divided into 4 subtypes: type 1, complete ureteral triplication (3 ureters with 3 orifices draining into the bladder); type 2, incomplete triplication, where 1 ureter bifurcates (3 ureters with 2 orifices draining into the bladder); type 3, trifurcated ureters (3 ureters with only 1 orifice draining into the bladder); and type 4, duplicated ureters, where 1 bifurcates in a reversed Y-shape (2 ureters with 3 orifices draining into the bladder). The incidence of these types in cases was 35%, 21%, 31%, and 9%, respectively, with the anomaly being more common on the left side. The case we report belongs to type 2, where 1 ureter has a normal orifice, while the other has closed off at both ends near the prostate, leading to the formation of a stone within the ureteral lumen.

The presence of ectopic ureters negatively affects the renal function. Owing to urinary obstruction and reflux urinary tract infections, function in the upper segment may be compromised, potentially leading to persistent urinary infections and stone formation.^[7]^ Clinical symptoms include recurrent urinary tract infections, perineal wetness, urinary incontinence, enuresis, or pain due to protrusion of the ectopic ureter. Some cases are asymptomatic and are often discovered incidentally during other examinations. In this case, the patient presented with significant symptoms of urinary tract infection and lumbar pain.

Most surgical interventions involve resection of the dysplastic renal segment and ureter. Liu et al^[8]^ reported a case in which 3 ureters were combined. Osipov et al^[9]^ also described a treatment plan utilizing laser incision for ureteral cysts. The type of surgical treatment depends on the presentation of the symptoms. The risk of renal failure is typically a decisive factor; however, when malformations of the ureter are present alongside vesicoureteral reflux, obstruction, ectopia, and recurrent infections, both the upper and lower urinary tracts can be treated simultaneously. In our patient, we achieved a satisfactory surgical outcome by resecting 2 segments of the malformed ureter and repairing the normal ureter.

Most patients with UT are asymptomatic; when symptoms occur, urinary tract infections are the most common. If ureteral duplication or triplication is suspected, detailed imaging is necessary. Patients with UT require individualized management and treatment strategies.

## Author contributions

**Conceptualization:** Longyuhe Yang.

**Data curation:** Longyuhe Yang.

**Formal analysis:** Longyuhe Yang.

**Resources:** Xuanfan Hu, Yunliang Zhao.

**Investigation:** Yueqiang Wang.

**Supervision:** Zhen Ma, Zhigang Zhang, Qiuyi Lu.

**Validation:** Yu Tian.

**Methodology:** Jianbing Yang.
